# Conformational Analysis of *Clostridium difficile* Toxin B and Its Implications for Substrate Recognition

**DOI:** 10.1371/journal.pone.0041518

**Published:** 2012-07-23

**Authors:** Rebecca Swett, G. Andrés Cisneros, Andrew L. Feig

**Affiliations:** Department of Chemistry, Wayne State University, Detroit, Michigan, United States of America; German Cancer Research Center, Germany

## Abstract

*Clostridium difficile (C. difficile)* is an opportunistic pathogen that can cause potentially lethal hospital-acquired infections. The cellular damage that it causes is the result of two large clostridial cytotoxins: TcdA and TcdB which act by glucosylating cytosolic G-proteins, mis-regulation of which induces apoptosis. TcdB is a large flexible protein that appears to undergo significant structural rearrangement upon accommodation of its substrates: UDP-glucose and a Rho-family GTPase. To characterize the conformational space of TcdB, we applied normal mode and hinge-region analysis, followed by long-timescale unbiased molecular dynamics. In order to examine the TcdB and RhoA interaction, macromolecular docking and simulation of the TcdB/RhoA complex was performed. Generalized Masked Delaunay analysis of the simulations determined the extent of significant motions. This combination of methods elucidated a wide range of motions within TcdB that are reiterated in both the low-cost normal mode analysis and the extensive MD simulation. Of particular interest are the coupled motions between a peripheral 4-helix bundle and a small loop in the active site that must rearrange to allow RhoA entry to the catalytic site. These extensive coupled motions are indicative of TcdB using a conformational capture mechanism for substrate accommodation.

## Introduction

One of the most common and serious hospital-acquired infections is *Clostridium difficile* (*C. difficile*), responsible for a suite of diseases collectively known as Clostridium difficile associated diseases (CDAD) [1], [2]. *C. difficile* typically affects patients undergoing antibiotic treatment for other infections, as it leaves the GI tract susceptible to colonization by this highly virulent pathogen due to the reduced protection by the normal gut microbiota [3], [4]. Currently, U.S. health care costs associated with treating CDAD are estimated to be between $750 million and $3.2 billion [4]–[8]. With the emergence of an epidemic strain that is both hypervirulent and more resistant to current therapies [9]–[11], costs will surely continue to rise, so new approaches to treating CDAD are needed.

*C. difficile* damages the intestines primarily through the action of two large protein toxins [3], Toxin A and Toxin B (TcdA and TcdB, respectively). These are members of the lethal subclass of large clostridial toxins [12]. The holotoxins are ∼300 KD and are comprised of four domains, each having a specific function related to cellular uptake and toxicity [13]. The CROP domain (Clostridial Repetitive Oligopeptide) helps to identify and bind to appropriate target cells by recognizing cell surface glycoproteins and inducing endocytosis [14]–[17]. The translocation domain is responsible for forming a transmembrane pore capable of passing the two remaining domains from the endosome to the cytoplasm [17]–[20]. A cysteine protease domain, activated by inositol hexakisphosphate in the cytoplasm, intramolecularly cleaves the cytotoxic glucosyltransferase (GT) domain from the holotoxin [21]–[23]. This last step is critical since at this point the GT domain is released into the cytosol where it can act on the RhoA, glucosylating residue T37 in the switch I region (or its equivalent S/T residue in the case of other Rho family members) [24]. Glucosylation of RhoA permanently inactivates it, causing defects in the cell-signal pathways that lead to cell rounding and ultimately apoptosis [24].

While one could develop new antibiotics to better target *C. difficile*, resistance is likely to be a major concern with any new agents. A potentially complementary approach to antibiotic therapy is to develop methods that target and neutralize the GT domain of the toxin [25]. Several steps in the etiology pathway could be targeted for inhibition, however this work focuses solely on the glucosyltransferase domain.

Several approaches are currently being used to therapeutically target TcdA and TcdB. Clinical studies are under way with humanized monoclonal antibodies that recognize and sequester the toxins, but this approach has some issues and will not be suitable for all patients [10], [26], [27]. Peptides and small molecules that recognize and inhibit toxin function are also being studied [28]. By better understanding the domain structures of the holotoxin, it will be easier to design or select molecules that disrupt their activity.

**Figure 1:**
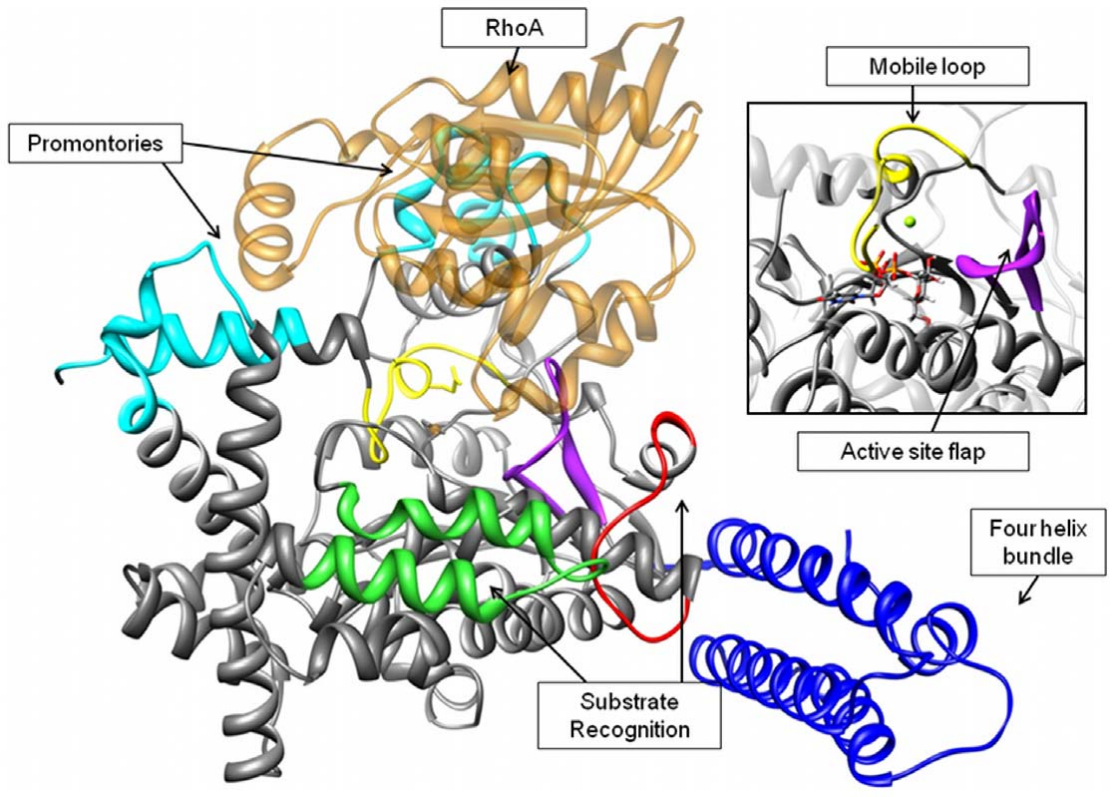
NM-RhoA docked complex. The most open normal mode conformation of the GT domain of TcdB is shown docked to the crystal structure of RhoA with its signaling loop in the “out” conformation. Relevant regions for discussion: The four helix bundle is shown in blue, the mobile loop containing the DXD motif is in yellow, the catalytic manganese is shown in black. Regions shown in green and red are involved in RhoA recognition. The β-hairpin shown in purple will be referred to as the active site flap. The upper regions in cyan are two flexible promontories unique to TcdB. RhoA is shown in transparent orange. **Inset: Active site, showing mobile loop and active site flap.**

The GT domain from TcdB (PDBID: 2BVL) was crystallographically characterized several years ago [29]. This domain was found to be a 543 amino acid domain that adopts a characteristic GT-A glucosyltransferase fold, and binds a catalytically-important Mn(II) ion. Previous studies comparing the *C. difficile* toxins to other glucosyltransferases, as well as extensive mutagenesis analysis on the toxins themselves, have identified a number of amino acid side chains critical for activity [30]–[32]. Figure 1 illustrates some of the important structural elements of TcdB that will be discussed later in the paper. A four helix amphipathic bundle comprising residues 1–87 (shown in blue) has been implicated in membrane association [33]; we will show that it is a key component in the large scale molecular motions exhibited by TcdB. Residues 510–522, shown in yellow, are part of a mobile loop which supports the catalytic manganese and includes a standard DXD motif. The two regions shown in cyan will be referred to as “upper promontories”. The function of these structural motifs is not yet understood, although they participate in a scissoring motion that will be described below. The beta hairpin shown in purple (residues 374–387) will be referred to as the active site flap and may have implications in catalysis and substrate recognition. The green region (residues 436–456) has been shown to be involved in recognition of RhoA by TcdB [34]. Finally, the red region (residues 483–497) shows motions that are highly correlated to those of the recognition site (residues 436–456) in our analyses [32], [35], [36]. Shown in transparent orange is RhoA, following docking.

A comprehensive understanding of the conformational space that TcdB is can occupy will better guide design of potential inhibitors. TcdB must pass through a pore to gain entry into the cell, therefore it is expected to have a flexible form to facilitate transient unfolding and refolding during translocation. Hinge region [37], [38] and normal mode analysis [39] were applied to determine the location and extent of the primary flexions. Both of these techniques have previously proven useful in determining the major motions attributed to well-studied systems, and give a fundamental impression of the overall motions one should expect to see in a flexible protein.

Long timescale unbiased molecular dynamics (MD) simulations may give insights both about the conformational space a protein occupies, as well as the mechanism of transition between those conformations. Additionally, the atomic scale detail in these simulations allows us to take a look at how large scale motions can have consequences in small regions, such as within an active site.

Understanding in a broad sense how TcdB moves and flexes both on its own and in contact with RhoA is expected to lead to better understanding of catalysis, substrate recognition and most importantly, drug design. The GT domain of TcdB has not yet been crystallized bound to substrates other than UDP-Glucose, and thus, nothing is known about the range of conformational space it can occupy, or what consequences binding to the RhoA protein might have. Recent evidence suggests that RhoA employs a conformational selection mechanism [40], rather than induced fit or lock and key. Thus, it is expected that a toxin targeting such a protein might have similar properties. Here we report normal mode and hinge region analysis, as well as long timescale molecular dynamics of TcdB. Additionally, macromolecular docking and long timescale simulation of the TcdB/RhoA complex was performed. Principal component analysis (PCA) and Generalized Masked Delaunay (GMD) analysis of the resulting conformations were used to help understand the conformational space TcdB occupies both alone and in complex with RhoA as well as the nature of the transitions between these conformational spaces.

## Results

### Flexibility studies and macromolecular docking

TcdB prefers to interact with RhoA in the GDP bound form based on binding studies [25]. Consequently the structure of RhoA bound to GDP was chosen (PDBID: 1FTN) for macromolecular docking [41]. Rigid body docking has been attempted previously, and was shown to result in only a rough approximation of a catalytic interface [29]. Our initial attempts at macromolecular docking used RosettaDock with Hex 4.5 as described in the methods section below. The resulting structure/energy plot did not display the cluster of low energy structures known as a “docking funnel” that is typically observed when a catalytically relevant docking conformation is revealed. Additionally, none of the structures placed the site of glucosylation near the active site of TcdB (data not shown).

Failure to form a docking funnel results when protein partners either do not bind, or undergo significant conformational changes before or during binding. Since we know the site of modification as well as a multitude of catalytic residues within the active site of TcdA/B, these initial docking attempts indicated that one or both partners must change conformation for the complex to achieve a suitable docking interaction. Therefore, we chose to apply normal mode analysis to this system to determine what major alternative conformations might be available to TcdB during substrate binding.

Using the crystal structure coordinates of TcdB as our starting point, water and substrates were removed and the resulting structure was submitted to the StoneHinge [39] and El Nemo [42] web servers. By analyzing both normal mode analysis and hinge region predictions, we expected to be able to define the major motions of TcdB as well as verify the locations of the flexions. The results from these two calculations showed good agreement in terms of predicting regions of high mobility and which residues provide hinge flexibility between the mobile regions (Figure S1, Movie S1).

While numerous normal mode conformations were docked to RhoA using the macromolecular docking protocol described below, the most open conformation from the El Nemo calculation gave the closest approach to a catalytically competent conformation. Figure 1 shows the orientation of RhoA with respect to TcdB following docking using the Hex and RosettaDock protocol. The switch region is oriented with Thr37 in a position to enter the active site, and there is good contact between residues both on TcdB and RhoA that have been shown to be critical for protein-protein binding [34]. However, the active site flap (Figure 1, shown in purple) is positioned to preclude close association between the two proteins. This interference in surface complementarity encouraged us to investigate the protein-protein binding interface.

The normal mode docked conformations showed improvement in binding over the crystal-crystal docked structures in proximity of the glucosylation site to the catalytic manganese. In the original docking, threonine 37 had a closest approach of 18 Å to the catalytic manganese. Subsequent docking to normal mode structures yielded a closest approach of 12.38 Å. A fully docked conformation might be expected to have a contact distance of between 7.1 Å and 7.7 Å based on comparison to several glycosyltransferases crystallographically characterized in the presence of UDP and an appropriate acceptor [43], [44]. Additionally, improvements were noted in the structure/energy plots (Figure S2). Overall complex energy is lower, and docked solutions are more tightly clustered. However, while the use of a normal mode structure improved the docking, none of the structures that were obtained were catalytically valid. It was concluded from these results that while the normal mode calculation represented some measure of the flexibility of the toxin, it was insufficient to model a conformation capable of glucosyltransferase activity–particularly with respect to the regions in and around the active site.

### Molecular dynamics and principal component analysis

To fully elucidate the interaction between these partners, MD simulations were set up as described in the methods section. Apo-TcdB and the structure of the normal mode conformation docked to RhoA (NM-RhoA) were simulated for a minimum of 150 ns. Our purpose in performing a full all-atom simulation was to determine what conformational changes occur in the TcdB/RhoA pair to allow binding when compared to TcdB in the absence of substrate.

In order to more effectively compare the conformational space occupied by TcdB through the MD trajectories, PCA was applied. PCA is useful in that it decomposes the complex motions of the simulation into the major types of movements that are observed across the entire trajectory. These can be observed as series of conformations varying in a single dimension.

**Figure 2:**
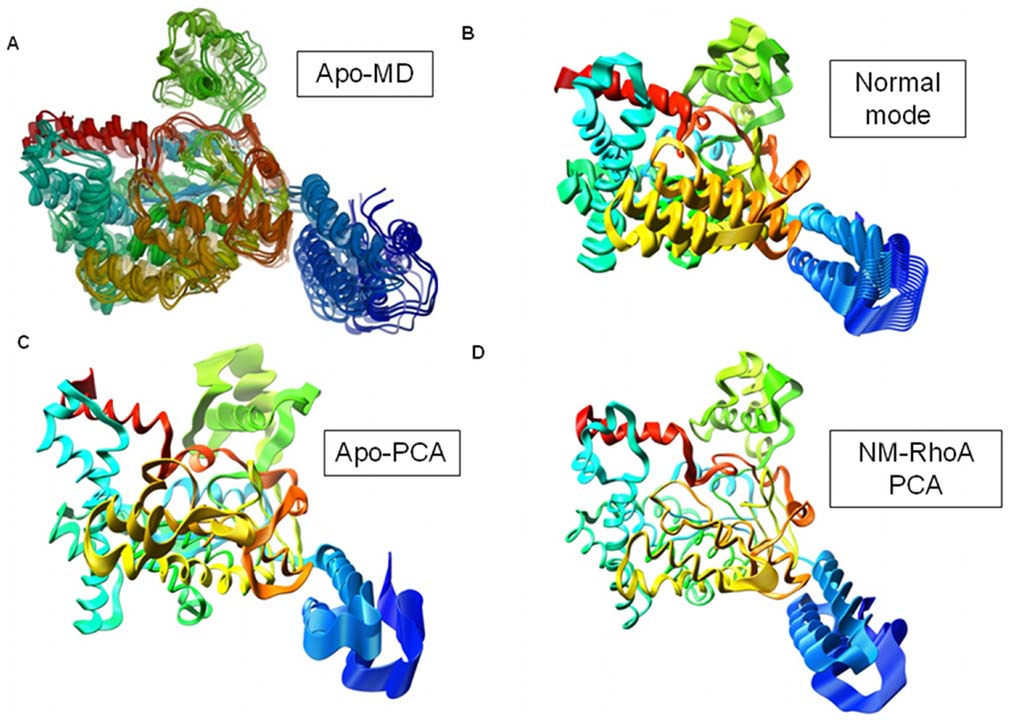
Comparison of general motile features of TcdB analyses and simulations. All structures are colored by rainbow per residue to allow better comparison between structures. A) Superposed frames representing various conformations in the Apo simulation, transparency indicates progression through the simulation. B) Normal mode structures of TcdB in the apo form. C) First principal component of the Apo simulation. Degree and direction of displacement are shown by broadened ribbons. D) First principal component of NM-RhoA simulation.

Analysis of the long MD simulations by PCA indicates that the principal component motions of the simulations echo the normal mode conformations as seen in Figure 2. Figure 2A shows a superposition of snapshots from the Apo molecular dynamics simulation. Figure 2B shows the results of the fundamental normal mode analysis. Figure 2C shows the first principal component extracted from the simulation of Apo-TcdB. Figure 2D displays the first principal component of the simulation of NM-RhoA. In normal mode analysis, MD, and PCA, the wagging motion of the four-helix bundle dominates, while the scissoring motion of the promontories is secondary. In each case, movement of these three regions affects the conformation of the highly flexible active site. The coupling of the motions of large peripheral structural elements of TcdB with highly specific rearrangements in the active site appears to be relevant to the process of substrate accommodation. Because normal mode analysis accurately predicts global protein movements in approximately 70% of cases [45], [46], agreement between these methods can be used as a measure of validation for the molecular dynamics simulations. In addition, it is apparent that in the NM-RhoA, the extent of flexibility is highly restricted (see Figures 2C and 2D). Qualitatively the motions remain quite similar, with the exception of movement in regions near the active site that will be discussed below.

Upon visual inspection the primary normal mode shows considerable similarity to the principal component motion of both the Apo-TcdB and NM-RhoA simulations throughout both trajectories, as can be observed by comparing panels B, C and D from Figure 2. It should be noted that the degree of motion is less pronounced when the protein is in contact with RhoA. This result is expected since there is a physical object impeding flexibility. Also, the second principal component, represented by the wagging of the upper promontories comprises a larger fraction of the variance in the Cartesian motions of both simulations (Figure S2).

The primary difference between the Apo-TcdB simulation and the NM-RhoA simulation occurs upon approach of RhoA to the catalytic center of TcdB. In the Apo-TcdB simulation, the active site flap (Figure 1 shown in purple) performs a repetitive back and forth motion, never completely obstructing the active site (Movie S2). During the course of the NM-RhoA simulation, the active site flap folds down directly over the TcdB active site, completely precluding access to the catalytic manganese (Movie S3). We interpret this behavior as indicative of the order of binding required for catalysis. In the absence of UDP glucose, the TcdB conformation required for successful RhoA is not accessible, and folding of the active site flap precludes close association. In the presence of UDP-glucose, this folding would not be possible, as the sidechains of the active site flap would run into the bound UDP-glucose. However, the similarities between the simulations indicate that the majority of the large-scale motion of TcdB has been captured, and this may be of interest to those designing RhoA mimics.

To assess improvements in the protein-protein interface following molecular dynamics, three structures were analyzed. One structure was selected as a representative frame from the most populated cluster throughout the simulation. The structure of the closest approach between Threonine 37 of RhoA and the catalytic manganese of TcdB was selected, as was the original normal mode docked structure; NM-RhoA. Table 1 lists the total number of interactions, number of hydrogen bonds, hydrophobic, ionic, aromatic-aromatic interactions, and cation-pi interactions. Hydrogen bonds are divided into main chain-main chain, side chain-main chain, and side chain-side chain interactions. The structures of both closest approach and most populated cluster both show improvement in the total number of interactions relative to NM-RhoA. Between the original docking and both MD structures, a shift from side chain-main chain interactions to side chain-side chain interactions occurs. No main chain-main chain hydrogen bonds were observed in any of the structures. A significant increase in ionic interactions is also observed relative to the original docked structures.

**Table 1:** Quantitative comparison of RhoA-TcdB contacts.

	NM-RhoA^a^	Closest^b^	Cluster^c^
Total interactions	33	45	42
H-bonds	20	24	20
MC-MC	0	0	0
SC-MC	19	4	4
SC-SC	1	20	16
Hydrophobic	8	5	7
Ionic	0	15	13
Aro-Aro	1	0	0
Cation-pi	4	1	2

a Structure of RhoA docked to the most open normal mode of TcdB.

b Structure of closest Thr37-Mn approach within NM-RhoA MD simulation.

c Structure of representative frame from the most populated cluster of the NM-RhoA MD simulation.

### Normal mode and molecular dynamics correlation

**Figure 3:**
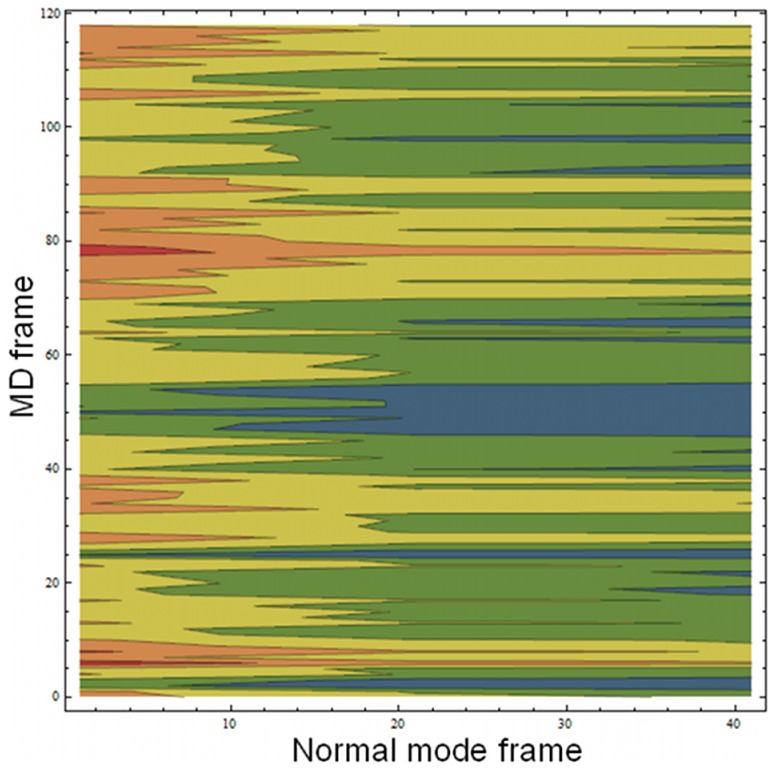
Heatmap of RMSD between simulation and normal mode conformation. Normal mode frame is on the X-axis, MD frame is on the Y-axis, and RMSD is shown as gradient from blue (low) to red (high). This arrangement allows observation of the correlated motions between the normal mode and the simulation. As the RMSD becomes low between the various normal mode structures and the MD simulations, occupation of the extremes of the normal mode conformations are observed. The periodicity seen in the plot can be interpreted as Apo-TcdB flexing through the range of normal mode conformations.

A heat plot was prepared to visualize the correlation between the normal mode and molecular dynamics trajectories. Figure 3 shows the RMSD from the normal mode structures across the dynamics trajectory. RMSD is plotted as a color scale while molecular dynamics trajectory frame and normal mode frame are on the y and x axes, respectively. This correlation results in a plot where the fluctuations in RMSD can be interpreted as the MD motions going in and out of phase with the normal mode conformations. For example, at roughly frames 25, 50 and 97 within the scaled trajectory, a low RMSD relative to the most open conformation of normal mode (Frame 41 on the x axis) is observed. This indicates that during the course of the molecular dynamics trajectory, Apo-TcdB exhibited a conformer similar to that of the normal mode structure, rebounded from that open conformation, and returned to the same open conformation later in the trajectory.

### Generalized Masked Delaunay and RMSF analysis

GMD analysis shows the rate of occurrence of significant events over the course of a molecular dynamics simulation. To create a time-dependent contact graph sensitive to large-scale conformational changes the GMD analysis performed utilized a Delaunay tetrahedralization. In this technique, a recrossing filter is applied to remove transient local positional changes that are the result of thermal motion. A plot of events per frame is generated following analysis, where the pattern of detected events in the context of contact making, breaking and total activity can be observed. In our analysis we observed no major folding events, and used the plots for comparative analysis of activity patterns.

**Figure 4:**
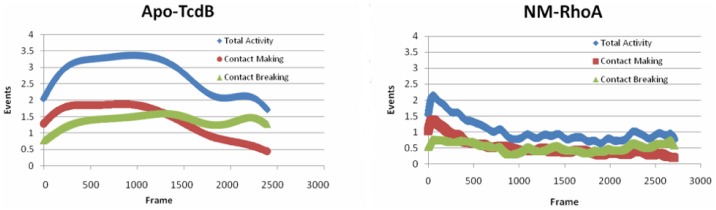
GMD analysis of both the Apo-TcdB and NM-RhoA simulations. This analysis plots events per frame through the course of the simulation. Total activity is shown in blue, contact making shown in red, and contact breaking in green. The event pattern indicates that while Apo-TcdB is flexing through its conformational space at a relatively constant pace, the NM-RhoA simulation undergoes a brief period of conformational rearrangement and then persists at a low level of activity through the rest of the simulation.

Figure 4 panels A and B are the results of a Generalized Masked Delaunay analysis across the molecular dynamics trajectories of Apo-TcdB and NM-RhoA respectively. Activity is plotted as events per frame, and is decomposed from total activity, shown in blue, to contact making (red) and contact breaking (green). The patterns of activity for Apo-TcdB compared with that of NM-RhoA are markedly different, with Apo-TcdB showing a relatively high level of activity throughout the simulation, while NM-RhoA very rapidly settles down and then exhibits a much lower level of activity throughout the simulation. This can be interpreted as a rearrangement followed by reduction of the available conformational space, or alternatively, a slowing of the transit between available conformations.

**Figure 5:**
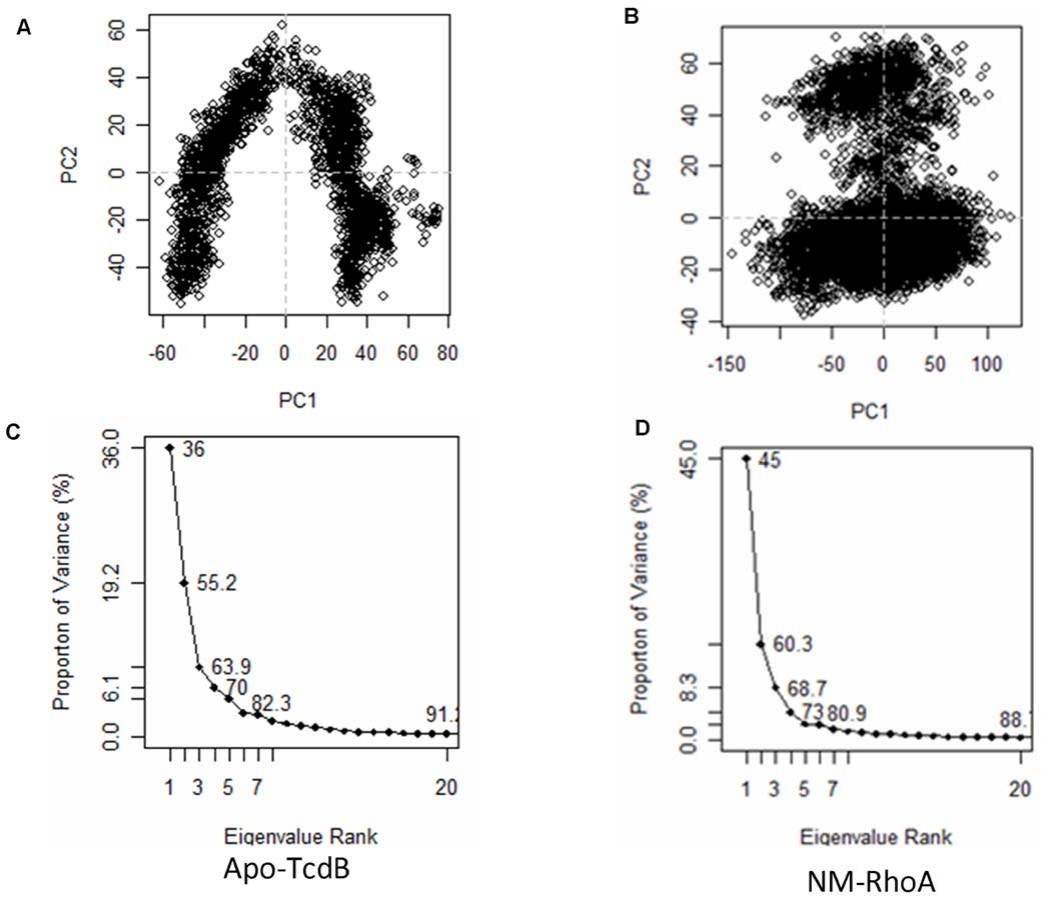
Principal component plots for the Apo-TcdB and NM-RhoA simulations. Panels A and B are the crossplots of the first and second principal components of the simulations. It is apparent in panel A that Apo-TcdB has a broad range of conformations available. Panel B shows three clusters of conformations observed during the NM-RhoA simulation, one of which is heavily populated. Plots of the proportion of variance to Eigenvalue rank indicate relative contributions of the lower order principal components. In the NM-RhoA simulation a slightly higher contribution from the primary normal mode is observed relative to the Apo structure. The slight decrease in the contribution from the second principal component in the NM-RhoA PCA analysis indicates that the scissoring motion of the upper promontories is less prevalent.

Throughout the Apo-TcdB simulation, the number of events per frame as shown in Figure 5 does not change dramatically, indicating a steady fluctuation between conformations rather than defined transitions. This can be interpreted as smooth flexion, rather than spontaneous and rapid conformational switches, providing support for the argument that the GT domain of TcdB utilizes a conformational selection mechanism to find its targets. It is likely that TcdB with bound substrate will have access to an alternative range of conformations that affects the movement of the active site flap when in contact with RhoA. While there is some overlap in conformational space of the Apo and bound simulations, the absence of UDP-Glucose precludes formation of a catalytic complex.

**Figure 6:**
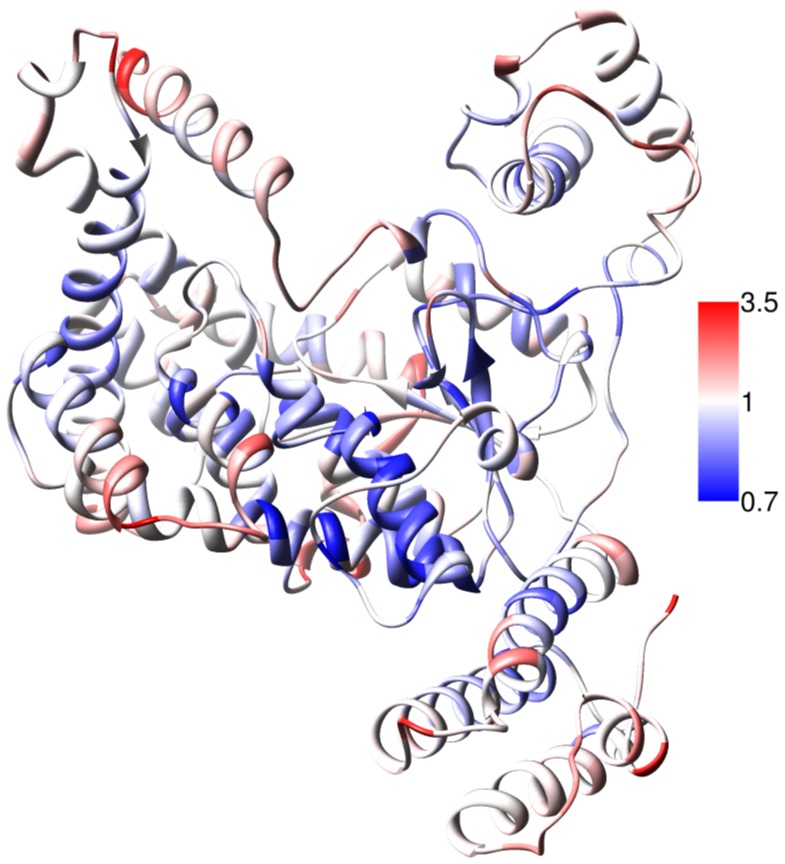
RMSF of Apo-TcdB. Rmsf was calculated across the Apo-TcdB simulation, and mapped onto the TcdB structure. Ribbons are colored by average atomistic rmsf per residue, from 3.5 Å (red) to 0.7 Å (blue). High flexibility is observed at the periphery of the protein, while the core of the four helix bundle and RhoA recognition site are stable. The active site flap and mobile loop reach rmsf values near 2 Å.

Over the course of the Apo simulation, major rearrangements have been observed in and around the active site. Both the mobile loop supporting the catalytic center, and the regions responsible for recognition of RhoA appear to be highly flexible. This flexibility is illustrated by the relative rmsf as shown in Figure 6, representing atomic freedom of motion over the time course of the simulation. It is expected that residues on a protein surface are quite flexible, while interior residues tend to be less mobile [47], [48]. The rmsf of TcdB ranges between 0.7 Å and 3.9 Å. In our simulation both mobile loops near the active site reach rmsf values of near 2 Å and thus undergo quite significant motions over time. The flexibility of the active site is unusual but understandable for this protein. Since the toxin must interact with a protein target well known for its conformational switch [41], flexibility near the active site would increase the ability to capture and glucosylate RhoA regardless of the conformation in which the switch is presented. Detailed analysis of the active site motions from MD simulations of TcdB in complex with UDP-Glc will be reported elsewhere (Swett, Cisneros and Feig, manuscript in preparation).

## Discussion

Application of normal mode analysis to the crystal structure of TcdB from *C. difficile* correctly captured the large-scale motions of this prototypical glucosyltransferase. The great degree of flexibility of TcdB is both expected and shown in evidence through normal mode analysis and molecular dynamics. A loose fold and considerable flexibility would be practical as the glucosyltransferase domain TcdB must, by necessity, thread through the membrane pore created by the translocation domain. The normal mode conformations bind RhoA moderately well while the crystal structure conformation of TcdB is completely incapable of forming a docked protein-protein complex. While the docking was unable to achieve a fully accommodated form where the toxin has Thr37 fully in the active site, this is a solid step towards determining the manner in which TcdB recognizes the Rho-family GTPases and excludes alternative G-proteins that might be structurally similar but which are not viable substrates.

In simulation, the conformations sampled between the Apo-TcdB and NM-RhoA bound structures are similar with respect to the primary normal modes. PCA plots in Figure 5, indicate that the NM-RhoA and Apo simulations are separately populated, with distinct conformational space occupancy. Taken together, this provides evidence for a conformational selection mechanism, which has been perturbed by Apo-Apo binding. In light of the dramatic alterations in the active site landscape through the course of the simulations it is possible that the presence of substrate may shift the conformation of TcdB towards a more suitable orientation for protein-protein binding.

Very recently, high resolution crystal structures for *C. difficile* Toxin A were reported, both alone and in complex with UDP-Glucose [49]. These proteins are highly homologous and catalyze the same glucosylation chemistry. Superposition of the TcdA structures shows considerable rearrangement of the active site in both the mobile loop, and active site flap. This has implications for the RhoA binding we observed. During the course of the NM-RhoA simulation, RhoA approach and active site flap orientation were correlated. In the absence of UDP-Glucose, the active site flap motions precluded close approach of RhoA to the catalytic center. In light of the rearrangements observed in the TcdA crystal structures, it is likely that conformational changes initiated by UDP-glucose binding are required before RhoA can be fully accommodated.

### Conclusions

We have performed unbiased long timescale simulations of TcdB from *C. difficile* both Apo and in contact with RhoA. Analysis on these trajectories included GMD, PCA, and comparison to motions observed in normal mode analysis. Large-scale flexibility was observed both in the presence and absence of a protein-binding partner without a catalytic binding event being observed. The dramatic rearrangement of the TcdB active site and the consequences for substrate binding point to the possibility that TcdB utilizes a conformational selection mechanism rather than lock and key, or induced fit binding.

It is logical that a protein that seeks out Rho GTP-ases would employ a conformational search mechanism, as Rho GTP-ases are known to employ conformational selection in their binding interactions both with small molecules and macromolecules

The exploration of this non-catalytic binding event has large implications for the kinetics of glucosyltransferase-substrate interactions. As anticipated, flexion in the active site alters substrate binding, and further study will elucidate the consequences of substrate binding on the conformational space available to TcdB. The combination of normal mode analysis, MD and GMD and PCA has been shown to be a very effective method for study of protein-protein interactions.

## Methods

Normal mode analysis of the toxin structures in question were performed via the El Nemo [39] web server and confirmed via hinge analysis using the StoneHinge [37], [38] hinge region prediction software. Docked conformations of the Apo-Toxin in contact with RhoA were generated using the RosettaDock [50] server using Hex 4.5 [51] for preliminary conformation generation, and systems were selected for simulation based on proximity to the catalytic binding site.

MD simulations were run using the charm27 [52]–[55] force field with the NAMD [56] suite of programs on the WSU rocks cluster. The canonical ensemble was maintained via periodic boundaries, Langevin dynamics and thermostat [42]. Simulation stability was verified by use of the trajectory analysis tools available with the VMD software [57]. Stability was monitored by energy and RMSD. Two systems were prepared and subjected to MD: Apo-TcdB and NM-RhoA.

The Apo-TcdB simulation includes only the TcdB structure, while the NM-RhoA simulation contains TcdB and RhoA in a putative docked conformation following protein-protein docking as described above.

The systems were solvated with TIP3P water, neutralized with counter ions and subjected to 1000 steps of conjugate gradient minimization and temperature ramped to 300K. The Apo-TcdB simulation contains 543 residues, 28,330 water molecules, and a total of 94,013 atoms. The NM-RhoA simulation contains 719 residues, 30,780 water molecules and a total of 102,970 atoms.

Frames from the trajectories were written every 1 ps. Apo-TcdB was simulated for 300 ns and NM-TcdB was simulated for 150 ns post minimization. The solvation box includes a 15 Å pad on each face of the box. Electrostatics were calculated using the particle mesh Ewald [58]–[60], and van der Waals were calculated with a nonbonded cutoff of 8 Å and a switching function between 7–8 Å. Results were analyzed by use of the GMD method, via the TimeScapes [61] software from the D.E. Shaw research group, as well correlation analysis manually handled by the Mathematica software [62]. For the purposes of the correlation analysis, a corkscrew interpolation was applied to the eleven original normal mode structures, resulting in a total of 41 normal mode structures. MD frames were selected evenly throughout the simulation, and pairwise RMSDs were calculated.

Analysis of the protein-protein interface was carried out across three structures using PIC [49]. Following clustering, a representative frame from the most populated cluster was selected, designated Cluster 1. The frame representing closest approach between Threonine 37 on RhoA and the catalytic manganese of TcdB was the second, and the NM-RhoA structure described above was the third. Hydrogen bond analysis was broken into two types, side chain-main chain interactions, and side chain-side chain interactions. Main chain-main chain interactions were looked for, but none occurred. Additionally, hydrophobic pairs, ionic, aromatic, and cation-pi interactions were tabulated.

## Supporting Information

Figure S1 **Hinge regions of TcdB.** Backbone is shown as a chain trace, hinge residues are represented as green spheres. Hinge regions are observed to occur between regions of flexibility in the normal mode analysis.
(TIF)

Figure S2 **Structure Energy plots generated following RosettaDock protocol.** Structure Energy is plotted against RMS from original docked complex guess. Panel A shows RhoA docking to the crystal structure of TcdB, and it can be observed that all energies are relatively high, and no cluster of low energy structures is observed. Panel B shows RhoA docked to the normal mode relaxed structure of TcdB. A reduction in docking energy is observed, and a few low energy regions are apparent. Of note is the improvement in docking when the normal mode structure of TcdB is utilized, indicating that flexibility in the face presented for docking may be a feature in TcdB's target recognition process.
(TIF)

Movie S1 **Normal mode conformations of TcdB.** TcdB is shown flexing through its primary normal mode, coloration same as in Figure 1. Flexion is observed in regions connected by the previously determined hinge regions, indicating that large scale conformational changes in this protein are likely.
(MP4)

Movie S2 **Conformational features of the Apo-TcdB molecular dynamics simulation.** The general range of conformations observed in the normal mode analysis are apparent over the 300 ns timecourse of the simulation. Additionally, motions near the active site do not result in permanent deformation, but a back-and-forth motion of the active site flap is observed. This wide range of flexibility is likely due to the absence of any substrate or binding partner, and is indicative of a conformational selection mechanism. The trajectory was visually smoothed by selecting frames at regular intervals. These were interpolated with a corkscrew algorithm with a linear interpolation rate over a period of 20 steps. The smoothed simulation is played from start to finish and reversed back to the beginning.
(MP4)

Movie S3 **Conformational features of the NM-RhoA molecular dynamics simulation.** The normal mode motions are again observed in this simulation, but to a lesser degree. Repacking of the NM-RhoA interface is observed, most significantly the folding down of the active site flap. This obscures the catalytic center of TcdB and does not allow close approach of the signaling loop of RhoA. Absence of UDP-Glucose is presumed to be the cause for this occurrence, as during a catalytic event it would occupy the space the side chains of the flap begin to enter. Regardless, it is interesting to note that the restriction of the conformational space indicates that upon protein-protein binding, the conformational space of TcdB is severely restricted, again pointing towards a conformational selection mechanism. A smoothed trajectory was used for visualization as described for Figure 3.
(MP4)

## References

[1] Pepin J, Valiquette L, Cossette B (2005). Mortality attributable to nosocomial Clostridium difficile-associated disease during an epidemic caused by a hypervirulent strain in Quebec.. CMAJ.

[2] Hookman P, Barkin JS (2009). Clostridium difficile associated infection, diarrhea and colitis.. World J Gastroenterol.

[3] Bartlett JG CT, Gurwith M, Gorbach SL, Onderdonk AD (1978). Antibiotic-accosicated, pseudomembranous colitis due to toxin-producing clostridia.. N Engl J Med.

[4] McFarland LV MM, Kwok RY, Stamm WE (1989). Nosocomial acquisition oClostridium difficile infection.. N Engl J Med 320: 204 210.

[5] Dubberke ER, McMullen KM, Mayfield JL, Reske KA, Georgantopoulos P (2009). Hospital-associated Clostridium difficile infection: is it necessary to track community-onset disease?. Infect Control Hosp Epidemiol.

[6] Dubberke ER, Wertheimer AI (2009). Review of current literature on the economic burden of Clostridium difficile infection.. Infect Control Hosp Epidemiol.

[7] Kyne L, Hamel MB, Polavaram R, Kelly CP (2002). Health care costs and mortality associated with nosocomial diarrhea due to Clostridium difficile.. Clin Infect Dis.

[8] McGlone SM, Bailey RR, Zimmer SM, Popovich MJ, Tian Y (2011). The economic burden of Clostridium difficile.. Clin Microbiol Infect.

[9] Huang H, Weintraub A, Fang H, Nord CE (2009). Antimicrobial resistance in Clostridium difficile.. Int J Antimicrob Agents.

[10] Merrigan M, Venugopal A, Mallozzi M, Roxas B, Viswanathan VK (2010). Human hypervirulent Clostridium difficile strains exhibit increased sporulation as well as robust toxin production.. J Bacteriol.

[11] Cookson B (2007). Hypervirulent strains of Clostridium difficile.. Postgrad Med J.

[12] Von Eichel-Streiber C SM, Thlestam M (1996). Large clostridial cytotoxins a family of glycosyltransferases modifying small GTP-binding proteins.. Trends Microbiol.

[13] Jank T, Aktories K (2008). Structure and mode of action of clostridial glucosylating toxins: the ABCD model.. Trends in Microbiology.

[14] Mathieu R, Lim J, Simpson P, Prasannan S, Fairweather N (2003). Resonance assignment and topology of a clostridial repetitive oligopeptide (CROP) region of toxin A from Clostridium difficile.. J Biomol NMR.

[15] Frisch C, Gerhard R, Aktories K, Hofmann F, Just I (2003). The complete receptor-binding domain of Clostridium difficile toxin A is required for endocytosis.. Biochem Biophys Res Commun.

[16] Ho JG, Greco A, Rupnik M, Ng KK (2005). Crystal structure of receptor-binding C-terminal repeats from Clostridium difficile toxin A. Proc Natl Acad Sci U S A.

[17] Giesemann T, Jank T, Gerhard R, Maier E, Just I (2006). Cholesterol-dependent pore formation of Clostridium difficile toxin A. Naunyn-Schmiedebergs Archives of Pharmacology.

[18] Pfeifer G, Schirmer J, Leemhuis J, Busch C, Meyer DK (2003). Cellular uptake of Clostridium difficile toxin B. Translocation of the N-terminal catalytic domain into the cytosol of eukaryotic cells.. J Biol Chem.

[19] Kaiser E, Kroll C, Ernst K, Schwan C, Popoff M (2011). Membrane translocation of binary actin-ADP-ribosylating toxins from Clostridium difficile and Clostridium perfringens is facilitated by cyclophilin A and Hsp90.. Infect Immun.

[20] Genisyuerek S, Papatheodorou P, Guttenberg G, Schubert R, Benz R (2011). Structural determinants for membrane insertion, pore formation and translocation of Clostridium difficile toxin B. Mol Microbiol.

[21] Egerer M, Jank T, Giesemann T, Aktories K (2008). Cysteine protease activity is responsible for autocatalytic cleavage of Clostridium difficile toxin A and B. Naunyn-Schmiedebergs Archives of Pharmacology.

[22] Shen A, Lupardus PJ, Gersch MM, Puri AW, Albrow VE (2011). Defining an allosteric circuit in the cysteine protease domain of Clostridium difficile toxins.. Nat Struct Mol Biol.

[23] Egerer M, Giesemann T, Jank T, Satchell KJF, Aktories K (2007). Auto-catalytic cleavage of Clostridium difficile toxins a and B depends on cysteine protease activity.. Journal of Biological Chemistry.

[24] Just I, Selzer J, Wilm M, von Eichel-Streiber C, Mann M (1995). Glucosylation of Rho proteins by Clostridium difficile toxin B. Nature.

[25] Ivarsson ME, Leroux J-C, Castagner B (2012). Therapien gegen Bakterientoxine.. Angewandte Chemie.

[26] Dawson LF, Valiente E, Donahue EH, Birchenough G, Wren BW (2011). Hypervirulent Clostridium difficile PCR-ribotypes exhibit resistance to widely used disinfectants.. PLoS One.

[27] Lanis JM, Hightower LD, Shen A, Ballard JD (2012). TcdB from hypervirulent Clostridium difficile exhibits increased efficiency of autoprocessing. Mol Microbiol..

[28] Abdeen SJ, Swett RJ, Feig AL (2010). Peptide inhibitors targeting Clostridium difficile toxins A and B. ACS Chem Biol.

[29] Reinert DJ, Jank T, Aktories K, Schulz GE (2005). Structural basis for the function of Clostridium difficile toxin B. Journal of Molecular Biology.

[30] Egerer M, Jank T, Giesemann T, Aktories K (2007). Involvement of cysteine residues in processing of Clostridium difficile toxins A and B. Naunyn-Schmiedebergs Archives of Pharmacology.

[31] Müller S, von Eichel-Streiber C, Moos M (1999). Impact of amino acids 22–27 of Rho-subfamily GTPases on glucosylation by the large clostridial cytotoxins TcsL-1522, TcdB-1470 and TcdB-8864.. European Journal of Biochemistry.

[32] Jank T, Giesemann T, Aktories K (2007). Clostridium difficile glucosyltransferase toxin B-essential amino acids for substrate binding.. Journal of Biological Chemistry.

[33] Geissler B, Tungekar R, Satchell KJF (2010). Identification of a conserved membrane localization domain within numerous large bacterial protein toxins.. Proceedings of the National Academy of Sciences of the United States of America.

[34] Jank T, Aktories K (2006). Chance of a single amino acid residue turns RhoD into a substrate of Clostridium difficile toxin B. Naunyn-Schmiedebergs Archives of Pharmacology.

[35] Jank T, Pack U, Giesemann T, Schmidt G, Aktories K (2006). Exchange of a single amino acid switches the substrate properties of RhoA and RhoD toward glucosylating and transglutaminating toxins.. Journal of Biological Chemistry.

[36] Jank T, Reinert DJ, Giesemann T, Schulz GE, Aktories K (2005). Change of the donor substrate specificity of Clostridium difficile toxin B by site-directed mutagenesis.. Journal of Biological Chemistry.

[37] Flores SC, Keating KS, Painter J, Morcos F, Nguyen K (2008). HingeMaster: Normal mode hinge prediction approach and integration of complementary predictors.. Proteins-Structure Function and Bioinformatics.

[38] Keating KS, Flores SC, Gerstein MB, Kuhn LA (2009). StoneHinge: Hinge prediction by network analysis of individual protein structures.. Protein Science.

[39] Suhre K, Sanejouand YH (2004). ElNemo: a normal mode web server for protein movement analysis and the generation of templates for molecular replacement.. Nucleic Acids Research.

[40] Grant BJ, McCammon JA, Gorfe AA (2010). Conformational selection in G-proteins: lessons from Ras and Rho.. Biophys J.

[41] Wei Y, Zhang Y, Derewenda U, Liu X, Minor W (1997). Crystal structure of RhoA-GDP and its functional implications.. Nature Structural Biology.

[42] Izaguirre JA, Catarello DP, Wozniak JM, Skeel RD (2001). Langevin stabilization of molecular dynamics.. Journal of Chemical Physics.

[43] Offen W, Martinez-Fleites C, Yang M, Kiat-Lim E, Davis BG (2006). Structure of a flavonoid glucosyltransferase reveals the basis for plant natural product modification.. EMBO J.

[44] Jiang JY, Lazarus MB, Pasquina L, Sliz P, Walker S (2012). A neutral diphosphate mimic crosslinks the active site of human O-GlcNAc transferase.. Nature Chemical Biology.

[45] Krebs WG, Alexandrov V, Wilson CA, Echols N, Yu HY (2002). Normal mode analysis of macromolecular motions in a database framework: Developing mode concentration as a useful classifying statistic.. Proteins-Structure Function and Genetics.

[46] Ma J (2005). Usefulness and limitations of normal mode analysis in modeling dynamics of biomolecular complexes.. Structure.

[47] Debrunner PG, Frauenfelder H (1982). Dynamics of Proteins.. Annual Review of Physical Chemistry.

[48] Petsko GA, Ringe D (1984). Fluctuations in Protein-Structure from X-Ray-Diffraction.. Annual Review of Biophysics and Bioengineering.

[49] Pruitt RN, Chumbler NM, Rutherford SA, Farrow MA, Friedman DB (2012). Structural Determinants of Clostridium difficile Toxin A Glucosyltransferase Activity.. J Biol Chem.

[50] Lyskov S, Gray JJ (2008). The RosettaDock server for local proteinprotein docking.. Nucleic Acids Research.

[51] Ritchie DWKDaVS (2005). High Order Analytic Translation Matrix Elements For Real Space Six-Dimensional Polar Fourier Correlations.. J Appl Crystl.

[52] Patel S, Brooks CL 3rd (2004). CHARMM fluctuating charge force field for proteins: I parameterization and application to bulk organic liquid simulations.. J Comput Chem.

[53] Patel S, Mackerell AD Jr, Brooks CL 3rd (2004). CHARMM fluctuating charge force field for proteins: II protein/solvent properties from molecular dynamics simulations using a nonadditive electrostatic model.. J Comput Chem.

[54] Brooks BR, Brooks CL 3rd, Mackerell AD Jr, Nilsson L, Petrella RJ, et al (2009). CHARMM: the biomolecular simulation program.. J Comput Chem.

[55] Sapay N, Tieleman DP (2010). Combination of the CHARMM27 force field with united-atom lipid force fields. J Comput Chem..

[56] Phillips JC, Braun R, Wang W, Gumbart J, Tajkhorshid E (2005). Scalable molecular dynamics with NAMD.. J Comput Chem.

[57] Humphrey W, Dalke A, Schulten K (1996). VMD: Visual molecular dynamics.. Journal of Molecular Graphics.

[58] Wang H, Dommert F, Holm C (2010). Optimizing working parameters of the smooth particle mesh Ewald algorithm in terms of accuracy and efficiency.. Journal of Chemical Physics.

[59] Darden T, York D, Pedersen L (1993). Particle Mesh Ewald–an N.Log(N) Method for Ewald Sums in Large Systems.. Journal of Chemical Physics.

[60] Essmann U, Perera L, Berkowitz ML, Darden T, Lee H (1995). A Smooth Particle Mesh Ewald Method.. Journal of Chemical Physics.

[61] Wriggers W, Kate A, Shan Y, Piana-Agostinetti S, Maragakis P (2009). Automated Event Detection and Activity Monitoring in Long Time-Scale Molecular Dynamics.. J Chem Theory Comput.

[62] Wolfram Research I (2010). Mathematica Edition: Version 8.0.. Champaign, Illinois: Wolfram Research, Inc.

