# Supplementary figures and images for: Conformational Analysis of Clostridium difficile Toxin B and Its Implications for Substrate Recognition
Source: PLoS One. 2012 Jul 23;7(7):e41518. doi: 10.1371/journal.pone.0041518 (PMC3402401; doi:10.1371/journal.pone.0041518)

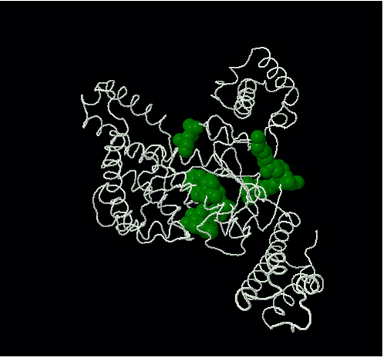

Supplement: Figure S1 — Hinge regions of TcdB. Backbone is shown as a chain trace, hinge residues are represented as green spheres. Hinge regions are observed to occur between regions of flexibility in the normal mode analysis. (TIF) [file pone.0041518.s001.tif]

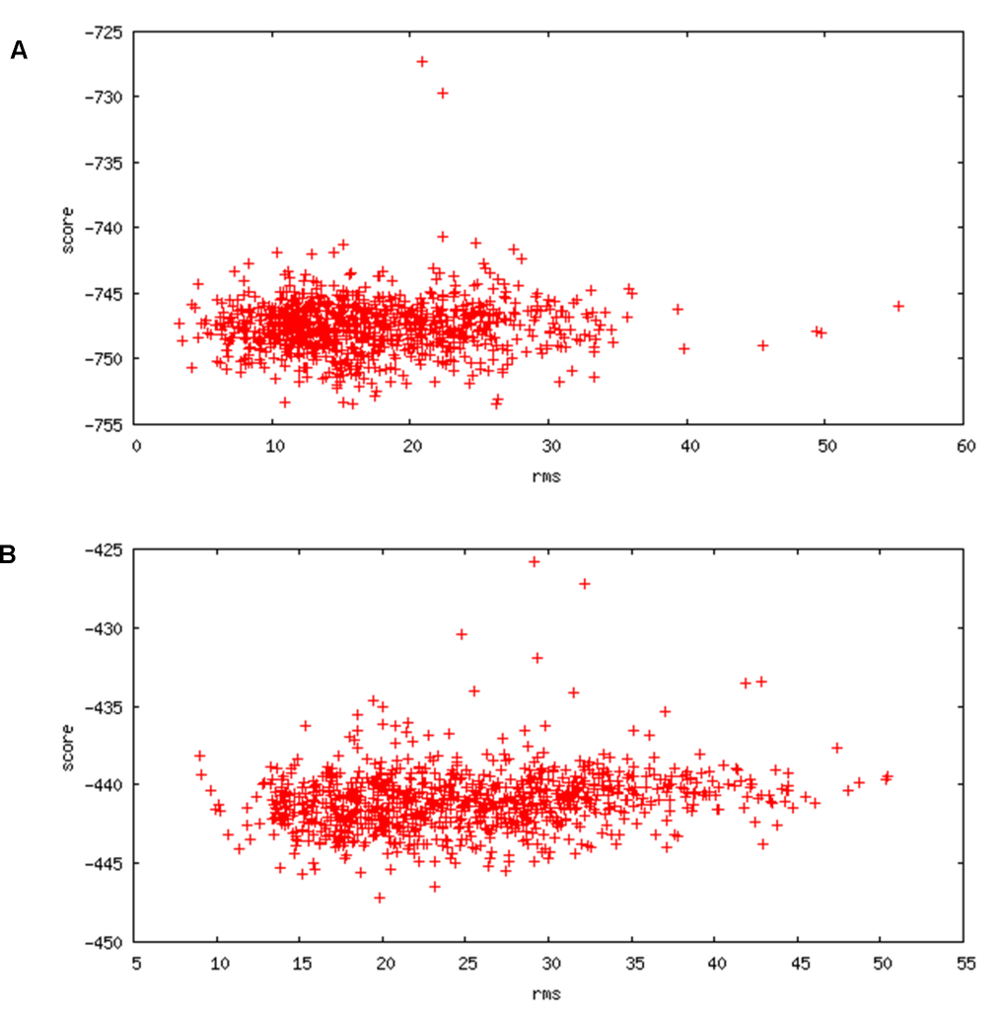

Supplement: Figure S2 — Structure Energy plots generated following RosettaDock protocol. Structure Energy is plotted against RMS from original docked complex guess. Panel A shows RhoA docking to the crystal structure of TcdB, and it can be observed that all energies are relatively high, and no cluster of low energy structures is observed. Panel B shows RhoA docked to the normal mode relaxed structure of TcdB. A reduction in docking energy is observed, and a few low energy regions are apparent. Of note is the improvement in docking when the normal mode structure of TcdB is utilized, indicating that flexibility in the face presented for docking may be a feature in TcdB's target recognition process. (TIF) [file pone.0041518.s002.tif]
